# The ribosome, (slow) beating heart of cancer (stem) cell

**DOI:** 10.1038/s41389-018-0044-8

**Published:** 2018-04-20

**Authors:** Amandine Bastide, Alexandre David

**Affiliations:** 0000 0001 2097 0141 grid.121334.6 IGF, CNRS, INSERM, Univ. Montpellier, F-34094 Montpellier, France

## Abstract

The ribosome has long been considered as a consistent molecular factory, with a rather passive role in the translation process. Recent findings have shifted this obsolete view, revealing a remarkably complex and multifaceted machinery whose role is to orchestrate spatiotemporal control of gene expression. Ribosome specialization discovery has raised the interesting possibility of the existence of its malignant counterpart, an 'oncogenic' ribosome, which may promote tumor progression. Here we weigh the arguments supporting the existence of an 'oncogenic' ribosome and evaluate its role in cancer evolution. In particular, we provide an analysis and perspective on how the ribosome may play a critical role in the acquisition and maintenance of cancer stem cell phenotype.

## Introduction

Accumulating evidence suggests that, far from being a consistent molecular factory, the ribosome may, in fact, be a critical variable in gene expression control. The existence of 'specialized ribosomes', characterized by unique composition and specific functions, is now solidifying^1,2^. Ribosome heterogeneity encompasses diversity in the composition of ribosomal proteins, plasticity in ribosomal RNA (rRNA) modifications and interaction with specific factors^1^. Such changes may impact ribosome function and activity, thereby exerting an important role in regulating spatiotemporal control of gene expression, both in normal and abnormal physiological functioning. In particular, correlation between congenital anomalies in either ribosome biogenesis or ribosome function (a.k.a. ribosomopathies) and cancer development has further refined the link between alteration of translation apparatus and cancer etiology^3^. Nevertheless, the mechanism by which an 'altered (oncogenic) ribosome' may promote tumor progression remains unclear.

The purpose of this review is to summarize and discuss the role of ribosome in cancer etiology and progression in the light of recent developments in ribosome biology. We start by providing a brief overview of current knowledge on mammalian ribosome, from its biogenesis to its essential role in fine-tuning gene expression. Then, we put forward arguments supporting the involvement of altered ribosome components in neoplastic process. We do not cover detailed regulatory mechanisms that steer translational control of the cancer genome, but this aspect has been thoroughly addressed in previous reviews^4,5^. Finally, on the basis of recent literature, we discuss whether the 'oncogenic' ribosome may fit within the current model of cancer evolution. In particular, we provide an analysis and perspective on how the ribosome may play a critical role in the acquisition and maintenance of Cancer Stem Cell (CSC) phenotype.

## The revisited ribosome

### With great 'power cost' comes great responsibility

The eukaryotic ribosome is a complex macromolecular machine made of 4 rRNA species and 80 ribosomal proteins (RPs)^6^. The mature ribosome is composed of 2 subunits, the small 40S ribosomal subunit containing the 18S rRNA and 33 RPs and the large 60S ribosomal subunit containing the 28S, 5.8S, and 5S rRNAs and 47 RPs. Ribosome biogenesis, one of the most complex and energy consuming process in the cell^6,7^, involves the coordinated work of the three RNA polymerases (RNA pol) and the assistance of more than 200 protein co-factors^8–11^. This stepwise journey commences in the nucleolus with the synthesis by RNA pol I of a large RNA transcript, the 45S pre-rRNA, which encodes three of the mature rRNA species (18S, 5.8S, and 28S rRNAs). The 5S rRNA is transcribed in the nucleoplasm by RNA Pol III and imported to the nucleolus. These rRNAs are then engaged in a series of modifications comprising nucleolytic processing steps and successive recruitment of RPs (transcribed by RNA pol II) in order to shape precursor ribosomal particles. Then, following further nucleoplasmic maturation steps, pre-60S and pre-40S ribosomes are eventually translocated to the cytoplasm where they undergo final maturation steps to form the mature 40S and 60S ribosomal subunits and achieve translation competence^8,10,12^.

The general process of translation takes place in the cytoplasm and is divided into three steps: initiation, elongation, and termination. A single mammalian cell expresses on average 10^5^–10^6^ cytoplasmic ribosomes at a given time and this number may vary by an order of magnitude of 3 to 10 across tissues^13,14^. This pool is carefully regulated by ribosome biogenesis and is adjustable to cellular needs^15–17^. Because 'free' ribosome availability is a limiting parameter in the process of translation^18^, any quantitative change in ribosome homeostasis may impact translation process^19,20^. First, mRNAs with low translation initiation rates are more 'sensitive' to variations in ribosome numbers in comparison with highly translated mRNAs^21^. Second, specific features of mRNA, such as upstream open reading frame (uORFs) and internal ribosome entry sites (IRES), may exert a growing influence on translation initiation frequency in response to ribosome scarcity^22–24^. Finally, ribosome density also impacts elongation rate: while small ribosome density along mRNA is not optimal, too many ribosomes may jam each other. Consequently, ribosome flux is maximized when the ribosomal density is halfway through the maximal possible density^25^.

**Fig. 1:**
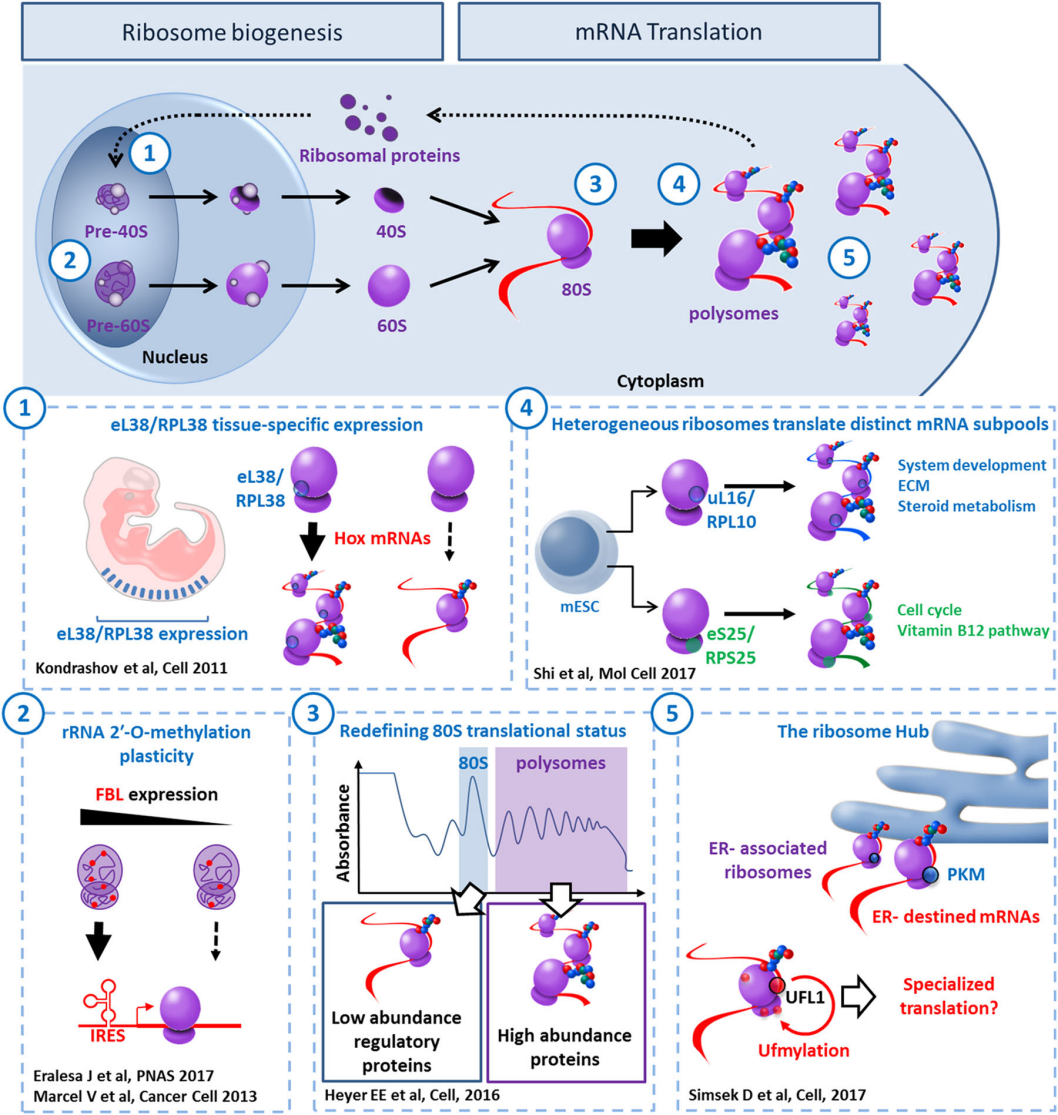
The multifaceted ribosome. The top banner gives an overview of ribosome biogenesis and mRNA translation. Along this process, each number refers to an illustration of ribosome heterogeneity and its corresponding impact on ribosome function. (1) In mouse, distribution pattern of mRNA encoding RPs varies significantly from one tissue to another. In particular, eL38/RPL38 expression is enriched in developing somites and neural tube. eL38/RPL38 is essential for translating specific Hox mRNAs that are involved in axial skeletal patterning. (2) During ribosome biogenesis, 2-O-methylation of the ribose is performed by the methyltransferase Fibrillarin (FBL). In human cells, any change in FBL expression may affect a repertoire of 2′-O-methylation sites showing plasticity. This translates into modulation of the intrinsic capabilities of ribosomes to engage IRES-containing mRNAs. (3) A recent ribosome profiling analysis preformed in *S. cerevisiae* revealed that, contrary to common belief, the vast majority of 80S monosomes were actively translating. Furthermore, besides translating short ORFs, uORFs, and Nonsense-mediated decay (NMD) targets, this ribosome population is specialized in synthesizing key regulatory proteins, such as transcription factors, kinases, and phosphatases. (4) Ribosome heterogeneity is observable at the sub-cellular level in murine embryonic stem cells. Differences in RPs composition bestow differential selectivity to translate mRNAs subpools. For instance, controlling the amount of uL16/RPL10A containing ribosomes participates in regulating expression of specific genes involved in metabolism, extra-cellular matrix (ECM) organization, cell cycling and development. (5) The surface exposed rRNA shell of eukaryotic ribosome provides an ideal interface for interacting proteins. Pyruvate kinase muscle (PKM) interacts with a sub-pool of endoplasmic reticulum (ER)-associated ribosomes and promotes translation of targeted mRNAs. UFL1 association with ribosome results in ufmylation of several RPs (uL16/RPL10, uS10/RPS20, and uS3/RPS3) and may be involved in subunit joining and contribute to transcript-specific translation

In addition to sheer number, the complex structure of the ribosome may provide another layer of translational control of gene expression. For decades the ribosome has been viewed as a mere automaton, a biological contrivance dedicated for catalyzing protein synthesis. This stereotypic view was greatly influenced by (A) the nature of its task, rather passive and mechanical, and (B) its remarkable efficiency to fulfill it: decoding mRNA at 5.6 codons per second^26^ with such accuracy^27^ necessitates both coordination and precision. A shift in this paradigm was initiated in 2002 with the prophetic 'ribosome filter hypothesis' from Mauro and Edelman^28^. Rather based on theoretical than experimental arguments, the authors challenged the monolithic perception of ribosomes as passive machines, and proposed that they might play an active control in gene expression regulation through 'mRNA filtering'. A decade later, the concept of 'specialized ribosome'^1^ emerged, soon supported by strong scientific arguments demonstrating heterogeneity in both RP and rRNA composition, as further substantiated in the following section (Fig. 1).

### The multifaceted ribosome

#### RPs expression

According to the 'RNA world' hypothesis, peptidyl transferase activity, responsible for peptide bond formation, may only require rRNA^29^. Therefore, what would be the use of so many RPs for the cell? Considering the complexity of ribosome biogenesis, the original role attributed to RPs was to stabilize rRNA structure in mature ribosome and play a chaperon-like role during ribosomal assembly^30–33^. Recent studies support this idea^34,35^. However, besides their nucleolar function in folding and processing rRNA, do all RP exert a role in mature ribosome activity as translation regulators or do some of them become 'dispensable' for this function? In other words, can mature ribosomes have heterogeneous composition? While most RPs seems to be essential for ribosome biogenesis^31^, several studies reported differential stoichiometry amongst the constituent core RPs in prokaryotes^36,37^ and eukaryotes^38,39^. In mammalian cells, RP expression varies greatly among tissues and even from one cell type to another^40–43^. This suggests that RPs expression pattern may evolve along cell differentiation, concomitantly with the acquisition of specialized functions. This is exemplified by eL38/RPL38 tissue-specific expression pattern during murine embryogenesis, which correlates with tissues that are affected by loss of function of this protein^42^. Likewise, human eL38/RPL38 expression pattern follows the same trend^44^. Another example of tissue-specific RP expression is uL16/RPL10 transcript, which is enriched both in murine epidermis and limb buds^45^. While uL16/RPL10 is among the few RPs to have a mammalian paralog, RPL10-like, these two RPs show differential expression, incompatible with reciprocal compensation^46^.

#### rRNA modifications contribute to ribosome heterogeneity

rRNA carries more than 100 chemical modifications, including base methylation, pseudouridylation, and ribose methylation at 2′-hydroxyl (2′-O-methylation)^47^. Besides their general role in stabilizing ribosome structure, these modifications cluster at key functional sites—such as peptidyl transferase and decoding centers—where they promote decoding efficiency and accuracy. The most abundant rRNA modifications are isomerisation of uridine into pseudouridine (Ψ) by pseudouridine synthases and H/ACA box small nucleolar RNAs (snoRNAs) and 2′-O-methylation of the ribose, performed by the methyltransferase Fibrillarin (FBL) and guided by C/D box snoRNAs^48–52^. Two recent studies have demonstrated the existence of rRNA 2′-O-methylation plasticity that would control the intrinsic capabilities of ribosomes to translate IRES-containing mRNAs^53,54^. In view of these recent developments, modifications in rRNA chemical pattern may well represent a new path toward functional specialization of ribosome.

#### Intracellular ribosome heterogeneity

Recent improvement in high-throughput sequencing and mass spectrometry technologies led to the development of innovative tools, such as ribosome profiling^26^ and selected reaction monitoring-base proteomics^55^, which contributed greatly to expose the existence of specialized ribosomes at the sub-cellular level, and expanded further the regulatory properties of translation machinery. First, contrary to common beliefs in the field, the vast majority of 80S monosomes are translationally active and may well represent specialized ribosomes involved in synthesizing key regulatory proteins, such as transcription factors, kinases, and phosphatases^56^. Second, ribosome heterogeneity has recently been demonstrated in a single cell type, defining subsets of ribosomes with heterogeneous RPs composition and endowed with differential selectivity for mRNA subpools^57^. Finally, the ribosome is a dynamic hub of interacting proteins that may enhance the potential diversity in ribosome composition, contribute to establish localized/specialized translation sites and connect translation machinery with specific cellular functions^58–60^.

Ribosome biogenesis and protein synthesis are involved in a considerable number of components and events and are highly regulated in order to efficiently respond to extrinsic demands. Multiple pathways are known to modulate ribosome biogenesis stoichiometry and assembly in order to prevent aberrant cell growth. In cancer cells, disruption of ribosome biogenesis and protein synthesis is associated with altered expression of key genes encoding translation initiation factors and proto-oncogenes such as mTOR, c-MYC, and RAS^61–63^. Dysregulation of ribosomal function, for instance following mutations in RP, is a field of rising interest, most particularly in the context of cancer evolution/progression, where a fundamental law prevails: 'survival of the fittest'.

## The oncogenic ribosome

### Translational control and tumor onset

Cancer is a generic term to designate hundreds of diseases endowed with hallmark traits, progressively acquired throughout tumor development: sustained proliferation, evasion of growth suppressors, resistance to cell death, replicative immortality, induced angiogenesis, immune evasion and activation of invasion, and metastasis^64^. Despite their complex nature, any given cancer shares as common feature an extensive and uncontrolled cell proliferation. This process is a direct consequence of enhanced protein synthesis which relies on ribosome biogenesis. For instance, the c-MYC transcription factor, often dysregulated in human cancers, increases protein synthesis by controlling the expression of multiple components involved in ribosome biogenesis and translation process^17,65^. Consequently, c-MYC activity enhances cell size and re-shapes nucleolar architecture. However, while the oncogenic potential of c-MYC relies on its ability to enhance protein synthesis^66^, aberrant cell growth and subsequent increased cell division is not sufficient to cause cell transformation. To counteract alteration of ribosome biogenesis, mammalian cells have developed tumor suppressor based surveillance mechanisms (e.g., TP53, PTEN, and RB1) that suspend cell proliferation in the event of uncontrolled ribosome production^67^. Known as the 'guardian of the genome', TP53 inhibits RNA pol I transcription machinery to block rRNA synthesis and maintain genomic and cellular homeostasis^68^. Disruption of this crucial monitoring mechanism—for instance through the acquisition of additional genetic lesions (secondary hits)—allows cancer cell to circumvent this limitation and maintain highly proliferative status.

Numerous studies have connected cancer genesis, evolution, and progression with dysregulated expression of individual RP (reviewed in^69^). Some reports have even correlated this alteration of RP expression with a poor prognosis^70–72^. However, the functional significance behind such variation, as well as the underlying RP-dependent regulatory mechanisms, remains elusive. The following sections intend to summarize actual arguments in favor of the existence of 'cancerous' alterations of the ribosome^73^ that would favor tumor onset.

### Alteration of RP expression

Solely based on the critical role of RPs in rRNA folding, any mutation in RPs or change in their relative expression may impact ribosome biogenesis and become deleterious—if not fatal—for transformed cell. Yet, changes in certain RP expression may facilitate cell transformation or cancer evolution. For instance, overexpression of RPLP1, a component of the 60S ribosomal P stalk, is sufficient to bypass replicative senescence in primary mouse embryonic fibroblasts and contribute to cell transformation in NIH3T3^74^. Likewise, in hepatocellular carcinoma, overexpression of eL42/RPL36A seems to promote disease progression, presumably by accelerating cell cycling program^75^. Lastly, overexpressed eL42/RPL34 promotes not only malignant proliferation, but also apoptosis resistance of non-small cell lung cancer cells^76^. By contrast, one may anticipate that any decrease in RP expression will impact ribosome biogenesis and subsequent levels of cytoplasmic mature ribosomes. Therefore, either mutation or downregulation of RP should repress tumor growth. Yet, in zebrafish, either loss or mutation of the wild-type allele of certain RP gene is sufficient to promote cancer development^77^. This paradox also applies for mammalian cells: in two independently induced murine cancers, uL6/RPL9 and uL24/RPL26 encoding genes behave as tumor suppressors, whose mutation or loss promotes tumor progression^78^. Other mouse models of RP haploinsuffciency are linked to tumor progression, such as uL5/RPL11 and eL22/RPL22 in mouse lymphoma development^79,80^ and uL18/RPL5 or eS24/RPS24 in mouse sarcomas^81^. uL18/RPL5 downregulation is also associated with breast cancer cell proliferation and tumor progression in transgenic mice and human tumor xenograft mouse model^82^.

When it comes to connect RP haploinsufficiency with elevated cancer incidence, the most striking example comes from ribosomopathies, a collection of disorders—congenital for the vast majority—in which genetic abnormalities trigger impaired ribosome biogenesis and function^83^. These syndromes result in specific clinical phenotypes that can be categorized as cellular hypoproliferative defects, often involving bone marrow failure and/or craniofacial or other skeletal defects. Remarkably, some of these diseases are associated with increased cancer risk, although the type and frequency vary significantly^83^. The best-studied ribosomopathy is named Diamond-Blackfan anemia (DBA) and characterized by bone marrow failure syndrome with a severe erythroid defect. Although this disease was first associated with recurrent mutations in eS19/RPS19 gene, further studies identified mutations or deletions in other RPs^84^. DBA patients who survive to adulthood have been reported to have a fivefold higher incidence of cancers, with a peculiar predisposition to colon cancer, osteosarcoma, and acute myeloid leukemia^85^ (AML). Other congenital disorders have been associated with defective ribosome biogenesis and cancer predisposition, including Schwachman–Diamond syndrome, Dyskeratosis Congenital (DC), cartilage hair hypoplasia, and Treacher Collins syndrome^86^. In the 5q syndrome, a subtype of adult myelodysplastic syndrome, the long harm of chromosome 5 is deleted, resulting in uS11/RPS14 haploinsufficiency and subsequent severe refractory anemia. It is not our aim here to give an extensive overview of ribosomopathies and for more in depth description we refer to excellent reviews^84,87^.

### Changes in rRNA expression and modifications

The role of rRNA expression and modifications in cancer progression is slowly emerging. Recent studies have correlated increased rRNA expression with cancer development in prostate and cervical cancer^88,89^. In colorectal cancer, high expression of the pre-45S rRNA promotes G1/S cell-cycle transition and is associated with poor prognosis^90^. Along the same lines, hypomodification of rRNA has been described in cancer and other diseases^3,91–93^, as well as in a rare ribosomopathy named X-linked Dyskeratosis Congenital (X-DC). X-DC is the most common and severe form of DC, and is consistently associated with mutations of the DKC1 gene^94,95^. DKC1 encode Dyskerin, an evolutionarily conserved enzyme involved in rRNA pseudouridylation of ~100 specific sites in rRNA. These modifications fine-tune rRNA folding and ribosome structure, enabling adjustment of ribosome-ligand (tRNA, mRNA) interactions^96^. Consequently, DKC1 mutation may impair both ribosome biogenesis and function. Alterations in rRNA 2′-O-methylation patterns are also involved in cancer evolution. In breast cancer, TP53 inactivation triggers FBL overexpression and subsequent changes in rRNA methylation landscape. This results in impaired translational fidelity and increased translation of IRES-containing mRNAs^54^. High level of FBL is generally associated with poor survival in primary breast tumors and was also reported in primary and metastatic prostate cancers and in squamous cell cervical carcinoma^97–99^.

### 'Ribosome-related signature' in cancer

Quite often, mutation or alteration of a single ribosomal component triggers global changes of ribosome-related genes’ expression. Although such changes may reflect a mere necessity to sustain high protein synthesis rate and rapid cell proliferation, they may hold prognostic or predictive values. For example, translational profiling analysis of chronic lymphocytic leukemia (CLL) patients led to the identification of a 'ribosome-related translational signature' comprising RPs, translation initiation factors, and DKC1^100^. The authors connect decreased DKC1 expression observed in CLL with reduced synthesis of certain RPs, which in turn would promote translatome alteration and the acquisition of an aggressive phenotype. Following the same trend, a ribosome-related signature associated with proliferative advantage and poor prognosis has been identified in NOTCH1-mutated CLL^101^, though the precise mechanism connecting NOTCH1 signaling with ribosome biogenesis remains unknown. Beyond its prognostic value, the identification of a 'ribosome-related signature' may also hold therapeutic potential. In breast cancer, miR-7641 targets uS9/RPS16 which, in turn, impact the expression of a subgroup of 10 ribosome-related genes^102^. The 'ribosome-related signature' thereby arising negatively correlates with patients’ survival status. Therefore, the authors suggest targeting miR-7641 in order to readjust RP expression and improve patient outcomes.

## The versatile ribosome

By definition, Darwinian evolution is based on the natural selection of small, blind, inherited genetic variations that increase the individual’s ability to compete, survive, and thrive. Cancer is a blatant example of such process: clonal evolution is driven by mutational changes that provide a fitness advantage relative to other clones within the same niche, thereby promoting selective clone expansion. Despite its role during the early stage of carcinogenesis, can alteration of ribosome function participate in cancer evolution? In this section we explore the evidence for a direct link between ribosome dysfunction and cancer progression in the context of the two current models of cancer evolution: the stochastic model and the hierarchical model.

### Ribosome alteration drives stochastic evolution

**Fig. 2:**
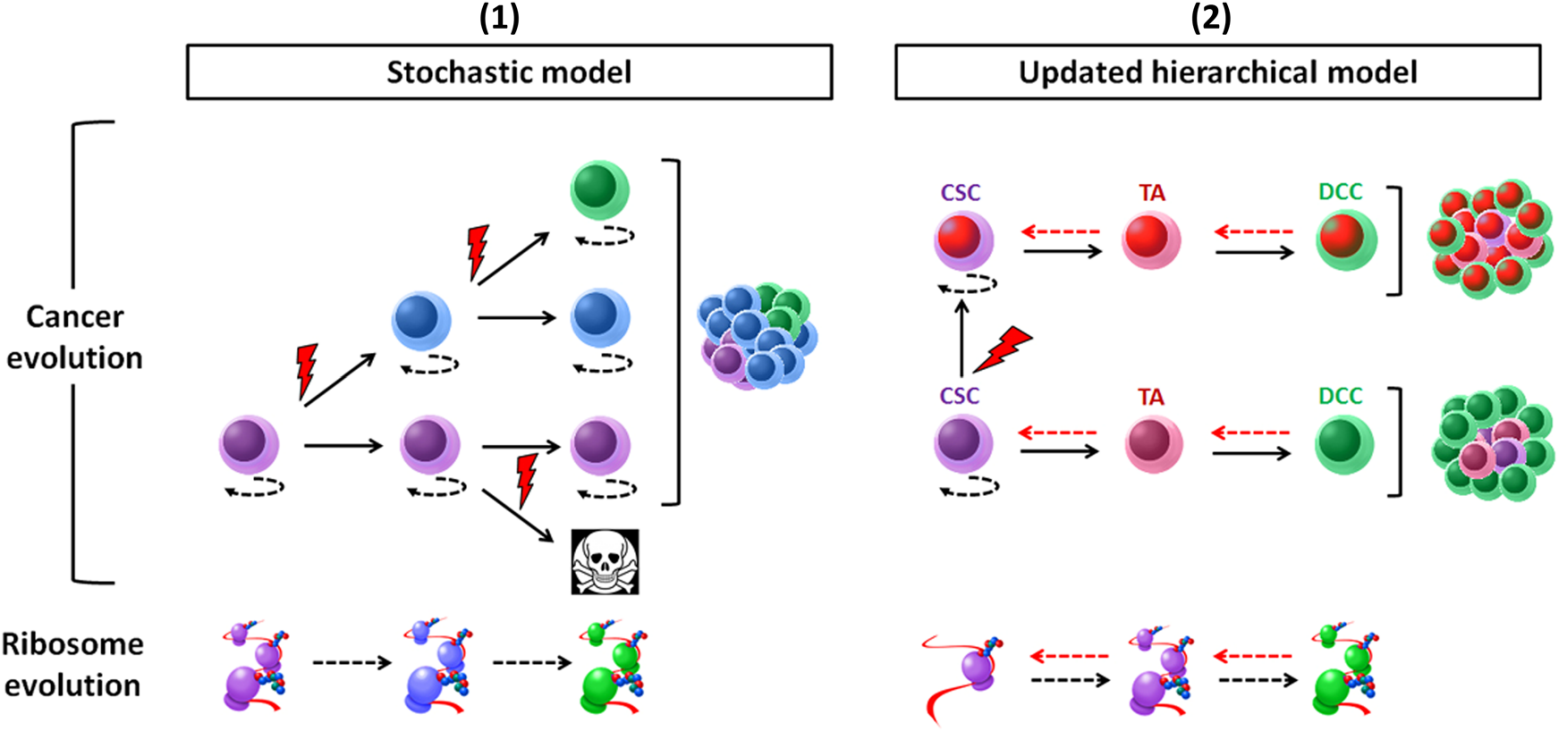
Ribosome and tumor evolution. (1) The stochastic model (left panel) assumes that every cell within the tumor has an equal likelihood to trigger tumor initiation and progression. Each clone harbors a different color. Clonal evolution is guided by intrinsic factors, in particular driver mutations (red lightening), such as somatic mutation of RPs. Deleterious mutations can also occur and lead to initiation of programmed cell death. Along this process, somatic mutations and environment constraints can change the structure and function of the translation machinery. (2) The hierarchical model (right panel). According to this model, cancer is organized in a hierarchical structure that partially resembles the tissue of origin. On the apex of this organization, CSC holds the tumorigenic potential and can differentiate into transient amplifying cell (TA), then differentiated cancer cell (DCC). In this illustration, CSCs harbor low translation activity. The differentiation process is accompanied with increased protein synthesis and may also drive ribosome 'evolution'. Cell plasticity (red dotted arrow) can reconcile both models. In this updated concept, cancer can be driven by more than one dominating clones which may display hierarchical organization

In the 'stochastic model', every cell within a tumor is equally likely to trigger tumor initiation and progression (Fig. 2). This process is guided by the interplay between advantageous or 'driver' lesions, neutral, or 'passenger' lesions, deleterious lesions, and changes in the tumor microenvironment that may alter the fitness effects of those lesions. A good driver is defined by specific features: reproducible and significant observation in multiple neoplasm (beyond background level), type of mutation, and association with clonal expansion. By opposition, a deleterious lesion is expected to be detrimental for cell growth and discarded by negative clonal selection process. Ribosome alteration can arise following somatic mutation of RP gene or as an indirect consequence of changes in ribosome-related genes' expression.

#### Cancer associated somatic mutations of RP

Recently, genome-wide analysis of tumor samples with next generation sequencing technologies revealed frequent somatic defects in multiple RP genes. Do these somatic mutations 'drive' tumor progression? A recent study identified six ribosomal protein genes as potential cancer drivers in five different cancer types: uL18/RPL5, uL5/RPL11, uL23/RPL23A, uS7/RPS5, uS10/RPS20, and uS2/RPSA^82^. Among them, uL18/RPL5 was the strongest candidate and most commonly mutated in human cancer, located at a significant peak of heterozygous deletion and either deleted or mutated in 11% of glioblastoma, 28% of melanoma, 34% of breast cancer patients and more than 20% of advanced multiple myeloma cases^82,103^. In T-cell acute lymphoblastic leukemia (T-ALL), somatic mutations and deletions of RP encoding genes have been reported in about 20% cases, the most frequent ones on uL16/RPL10 (8% of pediatric T-ALL cases) and eL22/RPL22 (10%), with rare defects in uL18/RPL5 (2%) and uL5/RPL11 (1,4%)^79,104,105^. Inactivating eL22/RPL22 mutations are also reported in 10% of gastric, endometrial, and colorectal cancer samples^106–108^. In melanoma, mutations in eS27/RPS27 5′ untranlsated region (5′UTR) is reported in 10% cases^109^. Finally, in aggressive CLL, somatic missense mutations of uS19/RPS15 occur in 10–20% of patients^110,111^. It is important to note that this list is certainly not exhaustive since some RP mutations may be only present in small cell subpopulations. A more complete picture will emerge with the democratization of single cell genome sequencing.

#### Translational mechanisms that may favor clonal expansion

The mechanisms by which alterations of ribosome biogenesis and activity confer competitive edge and drive both neoplastic development and clonal expansion are diverse. First of all, any quantitative change in ribosome concentration, even modest, may impact on the translation patterns and favor expression of specific mRNA to the detriment of others^87^. More specifically, a ribosomes shortage may primarily impact mRNAs that are inefficiently translated by the ribosome (low initiation rate), while translation of others could be unchanged or even improved^21,112,113^. This model is nicely illustrated by the impaired translation of GATA1 mRNA observed in DBA. GATA1 is an important erythroid transcription factor whose transcript harbors a highly structured 5′UTR. Consequently, GATA1 mRNA is inefficiently translated and particularly sensitive to ribosome deficiency that results from RP mutations^114^. Though this remains to be confirmed, alteration of GATA1 activity may participate in disease progression and clonal selection via its role in controlling cell proliferation and differentiation^115^.

Ribosomal alteration may also trigger translational dysfunction, which will have an impact on gene expression. Under some circumstances, such cascade of event will eventually confer selective advantages. For instance, the T-ALL-associated ul16/RPL10-R98S mutation triggers profound structural, biochemical, and translational fidelity defects that may drive cancer evolution through gene expression reprogramming^116^. In the same vein, ribosome modification may also promote differential translation of specific mRNA or the use of alternative translation initiation sites. In breast cancer, FBL expression alters rRNA 2′-O-methylation patterns, triggers changes in translational fidelity and promote cap-independent translation of IRES-containing mRNAs^54^. Hypo-pseudouridylation of rRNA is associated with impaired IRES‐dependent translational control of mRNA, including the tumor suppressors TP53 and p27Kip1 and the anti-apoptotic factors BCL-XL and XIAP^117,118^. Because of their heterogeneous nature, ribosome subpools may not be affected to the same extend by RP alterations. For instance, two RPs recurrently mutated in cancer, uL18/RPL5 and uL16/RPL10, are preferentially associated with monosomes^39^. As a result, these mutations might selectively impact a particular set of transcripts, which are preferentially translated by 80S monosome^56^.

In addition to their roles in ribosome biogenesis and protein production, RPs possess 'extraribosomal' functions that may be of relevance for understanding consequences of ribosome defects in cancer evolution and clonal selection. On one hand, expression level of certain RPs may promote cell growth and cancer cell proliferation. For instance, RPS13/uS15 overexpression promote cell-cycle progression of gastric cancer cells by downregulating the levels of p27(Kip1)^119.^ Noteworthy, RPS13/uS15, as well as RPL23/uL14, trigger cancer cell resistance to multiple chemotherapeutic drugs^120^. On the other hand, RP levels may also have inhibitory function: several RPs—in particular uL5/RPL11 and uL18/RPL5—trigger p53-dependent cell-cycle arrest, senescence, or apoptosis^121–123^. As another example of extraribosomal function, uL5/RPL11 can interact with c-MYC, an enhancing factor of ribosome synthesis^124^, and inhibit transcription of c-MYC target genes, including uL5/RPL11 itself as a negative feedback loop^125^. Several other extraribosomal roles of RPs have been thoroughly described in specialized reviews^126^.

### Ribosome and CSC

According to the hierarchical model, only a small subpopulation of the tumor, the CSCs, are capable of self-renewal and have potential to give rise to phenotypically diverse cells displaying limited proliferation and tumorigenic potential. Stochastic evolution and the CSC model are not mutually exclusive and can be reconciled by cell plasticity^127,128^. Phenotypic plasticity characterizes a population of cancer cells that have the capacity to interconvert between differentiated and stem-like states, through a continuum of cell fate specifications^129^ **(**Fig. 2**)**.

The CSC theory originates from early studies suggesting that cancer may retain some developmental programs underlying normal tissue organization^130–133^. The first solid evidence of CSC existence came from a study in 1997, where Bonnet and Dick isolated a set of stem cells from AML. These cells, once transplanted to immunosuppressed mice, were able to proliferate, differentiate, and to finally produce the same AML disease^134^. A decade later, the first CSCs in a solid tumor were identified in human breast cancer^135^. Since then, several studies reported identification of CSCs in most solid tumors such as gioblastoma^136^, melanoma^137^, osteosacarcoma^138^, head and neck squamous cell carcinoma^139^, pancreatic cancer^140,141^, lung cancer^142,143^, prostate cancer^144,145^, colon cancer^146,147^, and sarcoma^148^.

Several phenotypic markers have been proposed to identify and isolate CSCs from other tumor cells^149^, such as Lgr5^150,151^, CD133^152^, CD44^153^, and ALDH1A1^154,155^. However, none of them is a universal marker, i.e., capable of identifying and isolating CSC from multiple sources. Given such diversity of marker expression, CSCs are defined primarily by their functional capacities: self-renewal, tumor initiation, long-term tumor repopulation potential, and resistance to standard chemotherapies and radiation treatment^156^. The very existence of CSCs propels resistance to chemotherapy, disease progression, and relapse^157,158^.

#### Ribosome alterations and CSC phenotype

Recent reports connect alterations of ribosome components with acquisition or loss of CSC properties. In glioblastoma, increased expression of uS17/RPS11 and uS10/RPS20 is associated with stress resistant CSC phenotype and poor prognosis, while the precise mechanism remains unclear^159^. Along the same lines, silencing eL39/RPL39 expression was found to impact breast CSC abilities, such as self-renewal and metastatic potential, this time through inhibition of nitric oxide synthase signaling^160^. Analysis of lung metastases from a patient cohort confirmed the existence of a damaging mutation of eL39/RPL39 (AV14) in a significant number of samples. This mutation was undetectable in corresponding primary tumors, suggesting a clonal selection of a small cell sub-pool endowed with stem-like properties and showing increased metastatic potential^160^. Finally, eL22/Rpl22 haploinsufficiency in mouse promotes the development and the dissemination of T-cell Lymphoma by inducing expression of the reprogramming factor Lin28B^79^. Often associated with advanced disease across multiple tumor types^161^, Lin28B plays a significant role in self-renewal acquisition^162^ and has been proposed as CSC marker^157,163^.

Alteration of snoRNA, key players in rRNA modification and maturation, may also promote carcinogenesis^164^ and favor CSC phenotype acquisition. In particular, snoRA42 seems to be involved in invasiveness and metastasis of non-small cell lung cancer by regulating stemness of lung CSC. Although snoRA42 expression is elevated in CD133 + lung CSC, its depletion is associated with decreased self-renewal ability and suppression of tumor initiation in xenografted mouse^165^.

#### Low translation activity might confer CSC attributes

Besides the fact that a reduction in ribosome levels might be oncogenic in mammals, it might also promote stem-like phenotype. A functional genomics screen of mouse embryonic stem cells (ESCs), revealed that RP haploinsufficiency was inducing strong defects in embryoid body differentiation, while preserving self-renewal ability^166^. As pointed out by a recent study, occurrence of hemizygous RP deletions is common feature across human cancers, most particularly in concert with TP53 mutation^167^. Although it remains to be determined whether TP53 mutation precedes RP deletion throughout oncogenesis, these two alterations may synergize to promote CSC phenotype^168^.

Ribosome biogenesis and translational control play an important role in regulating proliferation, growth, and differentiation of embryonic stem cells in different organisms, such as *C. elegans*^169^, *Drosophila*^170–172^, and mouse^173,174^. In the hematopoietic compartment, protein synthesis in hematopoietic stem cells and multipotent progenitors is significantly lower in comparison with more differentiated cell types^43^. A recent study obtained similar results with epidermal stem cells and demonstrated that stem cells and their malignant counterpart—CSCs—harbor lower translation activity than committed cells^175^. One of the reason explaining such a trend could be that low protein synthesis may be essential for CSC metabolism. Yet, while the bulk of differentiated tumor cells relies on glycolysis rather than oxidative phosphorylation for ATP production and cell proliferation, no consensual metabolic pattern has emerged from studies comparing CSCs and non-CSCs (reviewed in^176,177^). Low translation rate may also be essential to maintain reduced proteasome activity, another hallmark of CSCs^178,179^. Indeed, the process of translation and nascent chain folding is intrinsically prone to errors and up to one-third of newly synthesized proteins do not achieve stable conformation, but rather are immediately degraded by the ubiquitin-proteasome system^180^. Any imbalance between the rates of synthesis and degradation of proteins may breach cell homeostasis and trigger cytotoxic accumulation of misfolded proteins. Further, maintaining low translation and degradation activities may prevent the presentation of tumor neo-antigens and contribute to CSC immune evasion abilities^181,182^. Finally, low ribosome activity may be essential to promote translation of a sub-pool of mRNA, which is essential for stemness maintenance. Therefore, any elevation of protein synthesis could trigger translational reprogramming and provoke cell differentiation, an appealing hypothesis supported by a recent study in *Drosophila* germline stem cell^170^.

It is important to note that, while most reports associate low translation activity with stem-like phenotype, this observation might be tissue-dependant. Indeed, molecules associated with protein synthesis have been shown to be upregulated in a breast cancer model of CSCs. The authors speculate that this enhanced protein synthesis drives CSCs proliferation leading to clonal expansion^183^.

#### Translational control of stress management

Stress conditions related to tumor microenvironment—such as hypoxia or nutrient starvation—favor low protein synthesis rate^184^ and sustain CSC phenotype^185^. Translational control is known to deliver fast and effective changes in gene expression levels^186^ and provide a reprieve for cells in order to forge an appropriate response and survive^187,188^. Because protein synthesis is a highly energy-consuming process, it is generally repressed in response to most stress conditions. However, this inhibition is not complete and concurs with selective translation of specific proteins involved in stress adaptation^184,187,188^.

The CSC phenotype is intimately associated with resilience and stress-response adaptation^189^, an observation which also holds for their physiologic counterpart^190^. Low ribosome content may influence alternative modes of translation initiation and promote translation of stress-protective proteins such as the transcription factor ATF4. Selective expression of ATF4 involves the existence of regulatory uORFs in the 5′-UTR, which capture scanning ribosomes and prevent translation of the main ORF in normal growth condition^191^. Once this inhibition is lifted, increased expression of ATF4 activates transcription of genes involved in amino acid metabolism, resistance to oxidative stress, and the proapoptotic transcription factor CHOP^192^. Remarkably, ATF4 is often upregulated in cancer and plays a role in self-renewal maintenance^193^. Cap-independent translation through the use of IRES elements is another key stress-response mechanism that may be enhanced by low ribosome content in CSC. This mechanism allows direct recruitment of ribosome to the mRNA in the 5′UTR through the IRES element and is facilitated by the binding of specific RNA binding protein called ITAF for IRES trans-acting factor. Numerous cellular ITAFs have been reported, among them the 'dispensable' ribosomal protein eS25/RPS25 which mediates c-MYC IRES dependant translation^194,195^. Overexpression of c-MYC is linked to hepatic CSC phenotype in a p53-dependent manner^196^ and, in glioma CSCs, c-MYC is required for proliferation, growth, and survival^197^. Other translational mechanisms account for stress resistance. To get more insight into the detailed mechanisms of translational control under stress, we recommend excellent reviews^184,187,188^.

Overall, while translational control in stem cells has been the subject of several reports, this topic has been superficially covered in their cancerous counterpart. Yet, a parallel may be drawn between the tremendous adaptation capabilities of CSCs and their low, flexible, stem-like, translation activity. We purposely didn’t cover metastasis in this review, although the metastatic process is another pertinent illustration of CSC resilience.

## Concluding remarks

For decades, the role of ribosome—and by extension translation—in gene expression control has been greatly underappreciated. A recent body of research has shifted our perception of ribosome from monolithic molecular automaton to specialized machinery, imbued with specific functions and regulating essential cellular processes such as cell growth, cell-cycle, metabolism, and cell migration. By contrast, ribosomal dysfunction has been intimately associated with cellular transformation and tumor progression. This bond is strengthened by the relationship between ribosomopathies and cancer susceptibility. However, several questions remain to be addressed, such as how hypoproliferative disorders can transition to cancer development, a disease characterized by extensive cell proliferation? One argument posits that RP defect creates the right circumstances to acquire and select rescuing secondary mutations by virtue of selection pressure^83^. Following this reasoning, it would be of interest to determine whether such a scheme—comprising initial ribosome dysfunction and compensatory secondary hits—would promote the emergence of clones imbued with CSC properties.

Cancer cells are consistently associated with dysregulation of ribosome biogenesis and increase in protein synthesis^3,198^. Therefore, several approaches have been envisaged, such as the development of therapeutic agents targeting key proteins involved in ribosome production and/or activity (e.g., EIF4A, EIF4E, RNA pol I, and mTOR)^199^. The use of antibiotics that interfere with ribosome activity has also been proposed. For instance, homoharringtonine (also named omacetaxine), a plant alkaloid that prevent the initial elongation step of protein synthesis, is indicated for treatment of chronic myeloid leukemia^200^. However, one must bear in mind that, since tumor is genetically heterogeneous, protein synthesis may vary from one clone to another and also according to differentiation state. Indeed, low protein synthesis rate, which contributes to the maintenance of undifferentiated state and self-renewal of CSCs^43,170,175^, may shield them from direct targeting of ribosome activity. L-Leucine has been recently administered to correct reduced translation in a DBA patient^201^. Similar treatment may increase translation rate in CSCs and promote their differentiation while stripping them from their stem-like abilities, including resistance to conventional therapies. Differentiation therapy holds great promise for cancer treatment^202^. Specifically, targeting CSC ribosome is another promising possibility, while it may necessitate a better understanding of its distinctive structural features.
